# Widely tunable Tm-doped mode-locked all-fiber laser

**DOI:** 10.1038/srep27245

**Published:** 2016-06-06

**Authors:** Zhiyu Yan, Biao Sun, Xiaohui Li, Jiaqi Luo, Perry Ping Shum, Xia Yu, Ying Zhang, Qi Jie Wang

**Affiliations:** 1 Center for Optoelectronics and Biophotonics, School of Electrical and Electronic Engineering & The Photonics Institute, Nanyang Technological University, 50 Nanyang Ave, 639798, Singapore; 2 Precision Measurements Group, Singapore Institute of Manufacturing Technology, 71 Nanyang Drive, 638075, Singapore; 3 School of Physics and Information Technology, Shaanxi Normal University, Xi’an 710062, P.R. China

## Abstract

We demonstrated a widely tunable Tm-doped mode-locked all-fiber laser, with the widest tunable range of 136 nm, from 1842 to 1978 nm. Nonlinear polarization evolution (NPE) technique is employed to enable mode-locking and the wavelength-tunable operation. The widely tunable range attributes to the NPE-induced transmission modulation and bidirectional pumping mechanism. Such kind of tunable mode-locked laser can find various applications in optical communications, spectroscopy, time-resolved measurement, and among others.

All-fiber lasers have attracted tremendous research interests due to the advantages of minimum alignment requirement, high energy efficiency, low maintenance, low sensitivity and wavelength independent to the temperature. Achieving widely tunable fiber lasers is of much interest because the emission wavelength can be tuned within a wide spectral range, which is useful for applications in spectroscopy, optical communication, optical instrument and system diagnostics, etc. This is convenient and cost-effective compared to using multiple lasers with different emission wavelengths. Comparing with tunable continuous wave (CW) lasers, tunable mode-locked lasers are irreplaceable light sources for time-domain and high-intensity applications. Examples are the sensitivity measurement of the absorption rate rather than the magnitude^1^, the non-linear spectroscopy and multiphoton microscopy, which require a high-intensity beam to stimulate the nonlinear phenomena^2^, and time-resolved photoluminescence^3^. Most tunable mode-locked fiber lasers are in the 1 μm and 1.5 μm regime and a few in 2 μm regime, in which Tm- or Ho-doped fiber is used as the gain medium^4^,^5^,^6^,^7^,^8^,^9^,^10^. Since Tm- or Ho- doped fiber lasers operate at the eye-safe regime and have broader emission spectra, they are particular important for medical and military applications having potentially a widely tunable range.

Various methods have been used to achieve tunable operation of all-fiber mode-lock lasers. Multimode fiber can work as a band-pass filter due to the multimode interference effects. The center wavelength of the band-pass filter, which depends on the length of multimode fiber, is fixed. In order to achieve tunability, some mechanisms are designed to change the effective length of multimode fiber^11^,^12^. Fiber taper also uses the modal interference with the evanescence field at different wavelengths, controlled by stretching the taper, to realize wavelength tunable operation^13^,^14^. Photonic crystal fiber induces mode coupling effect to make the propagating light resonant at certain wavelength. The tunability is by mechanically pressing the fiber to change the mode coupling, thus to change the resonant wavelength^15^,^16^. Fiber Bragg grating is another method that is commonly used. The shift of the wavelength is achieved when temperature, strain and/or pressure are applied to the fiber Bragg grating^17^,^18^,^19^. Another straightforward way is to use a tunable Fabry-Perot filter^20^, and the novel Fabry-Perot filters are based on microstructured fiber or micro-fiber^21^, micro-electro-mechanical system^22^, semiconductor modulator^23^, etc. Nonlinear amplified loop mirror (NALM) and nonlinear polarization evolution (NPE) are also used due to the designed cavity transmission modulation, the center wavelength of which is tuned by linear and nonlinear phase shift^24^,^25^,^26^,^27^. In Nelson’s work^28^, the tunable range is as wide as 104 nm, from 1798 to 1902 nm, with a Tm-doped fiber and an NPE cavity structure. It keeps the widest tunable range for mode-locked fiber laser for the time being, although some free-space components were used in the cavity. The widest tunable range of all-fiber mode-locked laser is about 50 nm by adopting a fiber taper filter^13^.

In this work, we have greatly extended the tunable range to 136 nm by combining NPE-induced transmission modulation technique and bidirectional pumping mechanism. Comparing to the tunable filters or gratings, most of which have mechanically moving parts and with the limited tunable range, the NPE technique can modulate the whole spectrum without limitation. The tunable range is then mainly dependent on the emission bandwidth of the gain medium, which is effectively increased by adopting the bidirectional pumping, rather than the unidirectional pumping. The cavity is in an all-fiber structure, and the tunable range is the widest in such kind of laser to the best of our knowledge.

## Results

**Figure 1:**
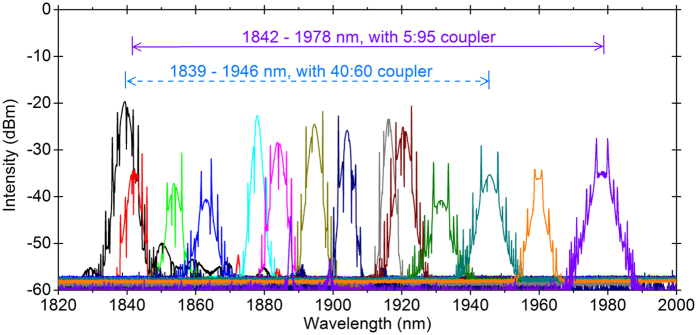
Tunable lasing emissions from 1839 to 1978 nm for Tm-doped mode-lock all-fiber lasers enabled by the nonlinear polarization evolution.

**Figure 2:**
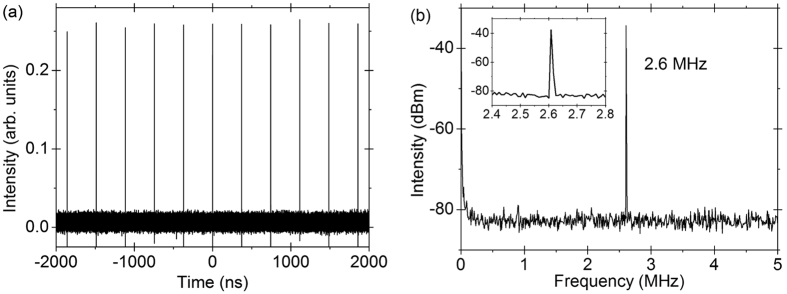
(**a**) The pulse train and (**b**) RF spectrum of the Tm-doped mode-lock all-fiber lasers.

The mode-locked laser emission is enabled by NPE effects and it appears when the pump power reaches 330 mW with 40:60 coupler. The output pulses are solitons with typical Kelly sidebands (the peak sidebands)^29^. The dips on the spectra are due to the water absorption^30^. By adjusting the polarization controllers (PCs), either rotating or squeezing, the emission wavelength can be tuned. The tunable range is 107 nm, from 1839 to 1946 nm with 40:60 coupler. When we changed the coupler with 5:95 splitting ratio the mode-locking threshold decreases to 300 mW, meanwhile the tunable range increases to 136 nm, from 1842 to 1978 nm, as shown in Fig. 1. The increased tunable range here is due to a lower threshold, to enable more mode-locking at the emission edge. The mode-locking usually works in the multi-pulse regime above the threshold, due to the nonlinear effects in a long cavity and the loss perturbation of the optical components. After the initiation of mode-locking and then decreasing the pump power slightly below the threshold, the mode-locking can work in the single-pulse regime. Further decreasing the pump power to 230 mw and 180 mW, for the 40:60 coupler and the 5:95 coupler cases, respectively, the mode-locking disappears. This is known as power hysteresis phenomenon. In single-pulse regime, the repetition rate is 2.6 MHz as shown in Fig. 2(a), corresponding to 80 m cavity length, which includes 70 m single mode fiber, 1.5 m Tm-doped fiber and fiber pigtails of other optical components. The pulse width is estimated to be several picoseconds as the output power is too low to measure the pulse width^31^ accurately. The RF spectrum in Fig. 2(b) shows that the pulse has a good signal-to-noise ratio of more than 40 dB. The 3-dB bandwidth of the RF spectrum is 1.35 kHz. The output power changes with the wavelength. With the 5:95 coupler, the laser near the center of the tunable range is with output power of ~1 mW, and the edge ones are with the output power of ~0.3 mW. With the 40:60 coupler, the laser near the center of the tunable range is with output power of ~3 mW, and the edge ones are with the output power of ~1 mW.

## Discussion

For the laser emits at short-wavelength and long-wavelength of tunable range, it usually contains some noise so that exhibits spectral content at other wavelengths. For the laser stability, the laser away from the edge of the tunable range (e.g 1860–1960 nm) is the most stable. It produces the stable pulse trains, keeps single-pulse emission over several hours, is insensitive to environmental perturbation, and has a broad pump power range to stay in single-pulse regime. While the laser near the edge of the tunable range is less stable, and the mode-locking is less insensitive to the perturbations.

**Figure 3:**
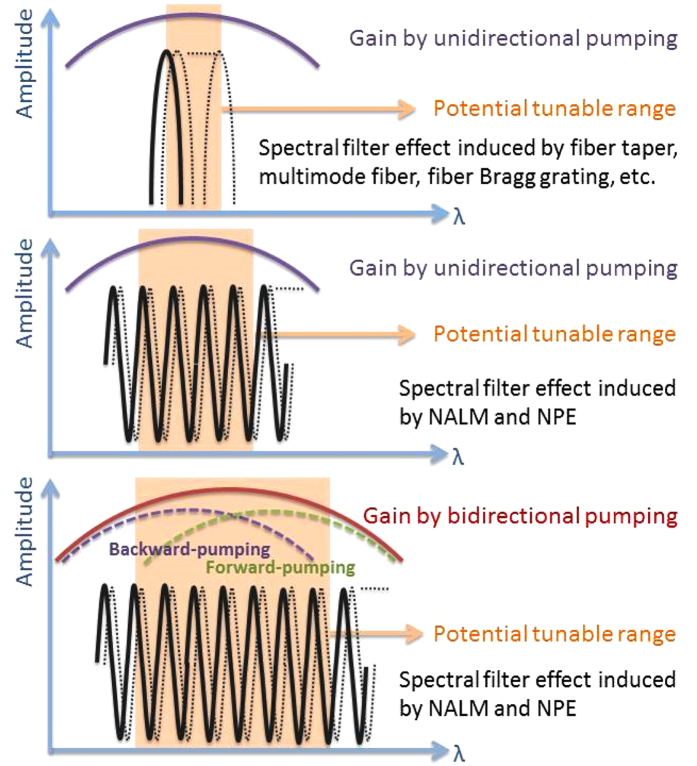
Schematic illustration of the effects of different pumping methods and spectral filters on the potential tunable range. The shaded areas describe the potential tunable ranges of each scheme.

To obtain a wide tunable range, the following two aspects are most important. The first is to broaden the gain bandwidth so that there is a potential wide range for wavelength tuning. The second is to form an artificial spectral filter with a tunable center wavelength, which acts as a wavelength selection element. In order to broaden the gain bandwidth, we adopted a bidirectional pumping scheme. The advantageous effect of this scheme was verified experimentally by measuring the amplified stimulated emission (ASE) spectrum under forward, backward and bidirectional pumping, which revealed that the bidirectional pumping resulted in the widest gain bandwidth. Experimental details are discussed in the following parts. For the artificial spectral filter, it can be formed by multimode fiber, fiber taper, photonics crystal fiber, fiber Bragg grating, Fabry-Perot filter, NPE, or NALM. Except NPE and NALM, the tunability of other components is due to the center-wavelength shifting of these spectral filters. The shifting is achieved by applying tension or stress to the component, which is limited by the variation of the physical structures. Up to now, the tunable range is several tens of nanometers. For NPE and NALM, the tunability is due to the periodic spectral filters stemmed from the NPE or NALM effect, which takes effects on the whole emission spectrum, not within a specific range. The wavelength tuning is achieved by changing the polarization state of the light inside the cavity, and details are in the following parts. Theoretically the tuning range can be as wide as the spectral range that above laser threshold. The combination of bidirectional pumping and NPE will increase the potential tunable range, the process is illustrated in Fig. 3. Last but not least, the overall cavity loss should be reduced to lower the laser threshold where the gain is smaller to let laser emit at more wavelength. Here we replaced the 40:60 coupler by a 5:95 coupler to reduce the cavity loss and, thus a broadband net gain (saturated gain minus cavity loss) will be formed to enable the broad laser emission.

**Figure 4:**
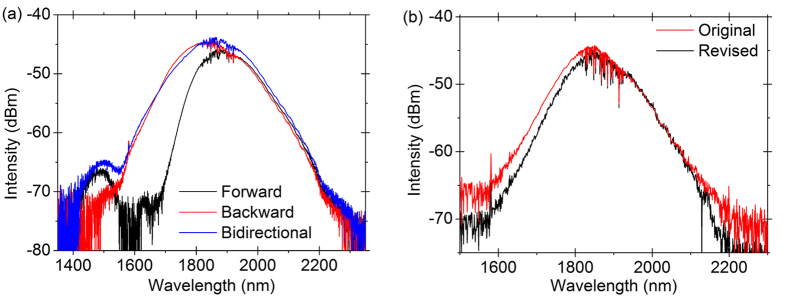
(**a**) The amplified stimulated emission (ASE) spectrum by bidirectional, backward, and forward pumping, under the same pumping power level. (**b**) The original and revised ASE spectrum. The revised one is with considering the wavelength-dependent loss of polarization-dependent isolator and coupler.

In order to have the widest gain bandwidth with our Tm-doped fiber, the bidirectional pumping method is adopted. Figure 4(a) is the measured ASE spectrum, which is an estimation of gain, for backward, forward and bidirectional pumping under the same pump power level. The spectral bandwidth of bidirectional pumping is the widest. Besides, the wavelength-dependent loss of optical components may affect the bandwidth of net gain. The wavelength-dependent loss of polarization-dependent isolator (PD-ISO) and coupler are investigated. For the PD-ISO, the working wavelength is 2000 nm and the working principle is based on Faraday rotator. As the rotation angle is wavelength-dependent, the loss induced by the rotation angle is also wavelength-dependent. Our laser emission wavelength is away from the working wavelength of PD-ISO, therefore the wavelength-dependent loss of PD-ISO should be taken into consideration. For the coupler, the coupling ratio will change over a wide wavelength range, which causes wavelength-dependent loss. The loss of PD-ISO and coupler are measured using a broadband light source. Figure 4(b) shows the revised ASE spectrum with considering the wavelength-dependent loss of PD-ISO and coupler. We can see that the wavelength-dependent loss of the two components takes minor effects on the bandwidth variance of the net gain spectrum.

We also recorded the tunable range of CW laser from the setup, the tunable range of CW laser matched with mode-locking. For further direction towards the wider tunable range, methods on engineering the net gain spectrum could be developed, to make the net gain spectrum wide and flat, thus to have a broad bandwidth supporting the tunable operation.

The NPE-induced cavity transmission modulation function is^32^:


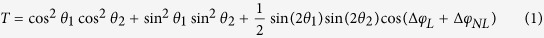


Where


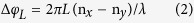



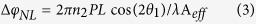



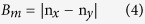


Δ*φ*_*L*_ is the linear phase delay, Δ*φ*_*NL*_ is the nonlinear phase delay, *B*_*m*_ is the strength of modal birefringence, *θ*_1_ is the angle between the fast axes of fiber and the polarization direction, *θ*_2_ is the angle between the fast axes of fiber and the orientation of polarizer inside PD-ISO, *L* is the fiber length, *P* is the instantaneous power of input signal, *n*_2_ is the nonlinear refractive index, *λ* is the operating wavelength, and A_*eff*_ is the effective mode area.

The mechanism for tunable operation is two-folded. Firstly, the cavity net gain is modulated periodically induced by NPE effect obtaining multiple peaks. Laser emits at the peak position of the net gain. With different cavity polarization states, laser tends to emit at one of the peak positions. Thus when tuning the PCs the cavity polarization states are changed, as well as at which peaks that laser tends to emit, so that the output wavelength of the laser is tuned. In this case, the tunable resolution equals to the separation of peaks in the transmission. With denser peaks the tunable resolution will be increased. 70-m single-mode fiber in the cavity is used to shorten the periodicity of the modulated transmission, thus to increase the number of peaks in the wavelength range of laser emission. However if too long fiber is used, too much transmission loss will be induced so that the mode-locked threshold will be affected. The calculated separation of peaks is around 10 nm^33^, which agrees with the experimental result and this is the tunable resolution. Secondly, only one peak of the modulated net gain is analyzed. It can be shifted by changing the light polarization angle *θ*_1_ and modal birefringence *B*_*m*_, depending on the equation^31^. *B*_*m*_ is changed when fiber inside the PCs has deformation by rotating or squeezing PCs. Thus the peak will ideally appear continuously at any position within one period. The tunable resolution in this case can be as small as possible. However because of the manual tuning operation we can achieve 0.5 nm tunable resolution.

## Conclusions

In this paper, we have demonstrated the tunable mode-locked all-fiber laser. The tunable range is 136 nm (from 1842 to 1978 nm), which is the widest in such kind of laser as far as we know. The NPE effect is responsible for activating the mode-locked operation and wavelength-tunable operation. Bidirectional pumping broadens the gain bandwidth so that increases the tunable range. This provides a simple and compact solution to widely tunable all-fiber lasers.

## Methods

### Experimental setup

**Figure 5:**
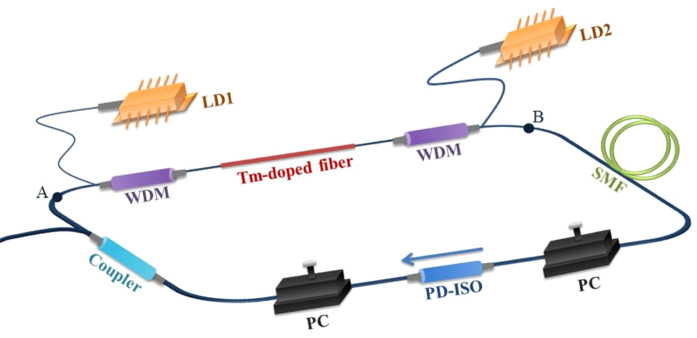
The experimental setup of the widely tunable Tm-doped mode-locked all-fiber laser. LD: laser diode. WDM: wavelength-division multiplexer. SMF: single-mode fiber. PC: polarization controller. PD-ISO: polarization-dependent isolator.

Figure 5 shows the experimental setup of tunable Tm-doped mode-locked all-fiber laser. A 1.5 m Tm-doped fiber (core/cladding diameter: 9/125 μm, Nufern) is bidirectional pumped by two single-mode laser diodes (LDs) with center wavelength at 793 nm. The maximum output power of the two laser diodes are 170 mW and 200 mW, respectively. The 793 nm pump light is coupled into the laser cavity by two wavelength-division multiplexers (WDMs), and 40% of the light inside the cavity is outputted through one coupler with 40:60 splitting ratio. A 70 m single-mode silica fiber and the 2 m polarization maintain fiber pigtails of the PD-ISO are used as the birefringent fiber. Two polarization controllers PCs and one PD-ISO induce the NPE effect. The dispersion of Tm-doped fiber and single-mode fiber (SMF 28) at 1900 nm are -71 and -67 ps^2^/km, respectively. With 1.5 m Tm-doped fiber and 78.5 m single-mode fiber including the fiber pigtail of the components, the net cavity dispersion is -5.37 ps^2^.

### Measurement method

The output spectrum and pulse trains are measured with optical spectrum analyzer (OSA) (Yokogawa, SN) and digital oscilloscope (Agilent) along with a fast photon detector (Newport, 12 GHz). When measuring the ASE spectrum, the laser cavity is disconnected at point A and B as shown in the Fig. 5. The fibers at point A and B are angle cleaved to avoid Fresnel reflection. The fiber at point A is connected to OSA to measure the ASE spectrum. When measuring the forward ASE, LD1 is off and the power of LD2 is set to 150 mW. When measuring the backward ASE, LD2 is off and the power of LD1 is set to 150 mW. When measuring the bidirectional pump effect, the total power of LD1 and LD2 is set to 150 mW. The pump powers for the three situations are the same.

### The extended results

**Figure 6:**
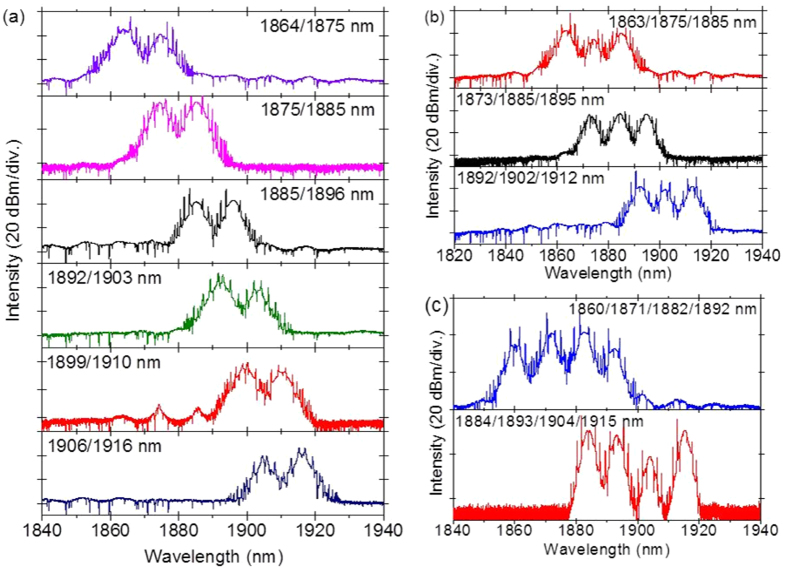
(**a**) Tunable dual-wavelength mode-locking from 1864 to 1916 nm. (**b**) Tunable tri-wavelength mode-locking from 1863 to 1912 nm. (**c**) Tunable four-wavelength mode-locking from 1860 to 1915 nm.

Besides the tunable single-wavelength mode-locking, tunable dual-, tri- and four- wavelength mode-locking can be also achieved by tuning the PCs, as shown in Fig. 6. The tuning range is 52 nm, from 1864 to 1916 nm for dual-wavelength mode-locking, and 49 nm, from 1863 to 1912 nm for tri-wavelength mode-locking, and 55 nm, from 1860 to 1915 nm for four-wavelength mode-locking, respectively. The multiwavelength emission is enabled when the net gain has multiple peaks, functioning as a periodic band-pass filter, and the optical power is equally distributed among two or three or four peaks, to reduce the mutual mode competition. This multi-peak net gain is formed by the periodic modulation of cavity transmission, induced by NPE effect. The detailed analytical work has been explained in our previous work^31^.

## Additional Information

**How to cite this article**: Yan, Z. *et al.* Widely tunable Tm-doped mode-locked all-fiber laser. *Sci. Rep.* **6**, 27245; doi: 10.1038/srep27245 (2016).
